# Association of sleep disorders with heart rate variability in children and adolescents with cystic fibrosis

**DOI:** 10.1590/1984-0462/2022/40/2020295

**Published:** 2021-09-01

**Authors:** Rodrigo dos Santos Lugao, Roberta Ribeiro Batista Barbosa, Pitiguara de Freitas Coelho, Fernanda Mayrink Gonçalves Liberato, Pâmela Reis Vidal, Roberta Barcellos Couto Olimpio de Carvalho, Roberta de Cássia Nunes Cruz Melotti, Márcio Vinícius Fagundes Donadio

**Affiliations:** a School of Sciences, Santa Casa de Misericórdia de Vitória, Vitória, ES, Brazil.; b Cystic Fibrosis Reference Center, Hospital Infantil Nossa Senhora da Glória, Vitória, ES, Brazil.; c Study Center on Sleep Medicine, Vitória, ES, Brazil.; d Laboratory of Pediatric Physical Activity, Centro Infant, Pontifícia Universidade Católica do Rio Grande do Sul, Porto Alegre, RS, Brazil.

**Keywords:** Cystic fibrosis, Sleep disorders, Heart rate variability, Hypoxemia, Pediatrics, Fibrose cística, Distúrbios do sono, Variabilidade da frequência cardíaca, Hipoxemia, Pediatria

## Abstract

**Objective:**

To assess the association of sleep disorders with the findings of heart rate variability (HRV) in children and adolescents with cystic fibrosis (CF).

**Methods:**

Cross-sectional study including children and adolescents aged six to 18 years with a clinical diagnosis of CF. Sociodemographic and clinical data were collected. Sleep disorders were evaluated using baseline nocturnal polysomnography. The autonomic nervous system (ANS) was evaluated through resting HRV.

**Results:**

A total of 30 individuals (11.2 years) with a mean forced expiratory volume in the first second (FEV_1_) of 62.7% were included. The respiratory disturbance index presented a median of 2.6 and obstructive sleep apnea syndrome (OSAS) was identified in 30%. In the HRV analysis, a mean standard deviation of all inter-beat (RR) intervals (SDNN) of 60.8±45.9ms was found. There was a significant correlation between the HRV low-frequency/high-frequency (LF/HF) global modulation index and the minimum SpO_2_ during sleep in patients with FEV_1_<60% (r=0.71; p=0.02). The prevalence of sleep disorders and HRV abnormalities was higher in individuals with lesser pulmonary function (FEV_1_<60%).

**Conclusions:**

The results indicate a weak correlation of sleep disorders (minimum SpO_2_) with HRV parameters (LH/HF) in children and adolescents with CF. When pulmonary function was reduced, a stronger correlation was found, highlighting the influence of disease severity. A high prevalence of ANS disorders, nocturnal hypoxemia, and presence of OSAS was also found.

## RESUMO

**Objetivo:**

Verificar a associação de distúrbios do sono com os achados de variabilidade da frequência cardíaca (VFC) em crianças e adolescentes com fibrose cística (FC).

**Métodos:**

Estudo transversal, incluindo crianças de 6 a 18 anos com diagnóstico clínico de FC. Dados sociodemográficos e clínicos foram coletados. Os distúrbios do sono foram avaliados por polissonografia basal noturna. O sistema nervoso autônomo (SNA) foi avaliado através da VFC em repouso.

**Resultados:**

Trinta indivíduos (11,2 anos) com média de volume expiratório forçado no primeiro segundo (VEF_1_) de 62,7% foram incluídos. O índice de distúrbios respiratórios do sono apresentou mediana de 2,6 e a síndrome da apneia obstrutiva do sono (SAOS) foi identificada em 30%. Na análise da VFC, foi encontrado um SDNN, desvio padrão médio de todos os intervalos RR (entre duas ondas R), de 60,8±45,9ms. Houve correlação significativa entre o índice de modulação global da VFC baixa-frequência/alta frequência (LF/HF) e a SpO_2_ mínima durante o sono em pacientes com VEF_1_<60% (r=0,71; p=0,020). A prevalência de distúrbios do sono e anormalidades na VFC foi maior em indivíduos com menor função pulmonar (VEF_1_<60%).

**Conclusões:**

Os resultados indicam uma correlação fraca dos distúrbios do sono (SpO_2_ mínima) com os parâmetros da VFC (LF/HF) em crianças e adolescentes com FC. Nos pacientes com função pulmonar reduzida, uma correlação mais forte foi encontrada, destacando a influência da gravidade da doença. Também foi encontrada alta prevalência de distúrbios do SNA, hipoxemia noturna e presença de SAOS.

## INTRODUCTION

Cystic fibrosis (CF) is an inherited autosomal recessive disease that compromises and limits the function of almost every organ and system in the human body. Among several complications, individuals with CF often present sleep disorders, including hypoxemia and obstructive sleep apnea syndrome (OSAS).^1^ ^,^ ^2^ Episodes of nocturnal hypoxemia have been related to worsening pulmonary function and, consequently, increased mortality.^3^ ^,^ ^4^ In addition, the decrease in the peripheral oxyhemoglobin saturation (SpO_2_) during the night has been associated with pulmonary hypertension, reduced sleep efficiency, and neurocognitive dysfunction during exacerbations.^5^ Ramos et al.^6^ demonstrated that 6% of 67 clinically stable children with CF presented hypoxemia during their sleep, whereas 26.9% of the children studied had significant desaturation (SpO_2_<85%). These events were also observed to be associated with worse clinical results, including the Shwachman-Kulczycki score, forced vital capacity (FVC), and forced expiratory volume in the first second (FEV_1_); and a higher proportion of rapid eye movement (REM) stage sleep.

Therefore, early evaluation and detection of sleep disturbances may contribute to a better quality of life and prognosis.^1^ ^,^ ^5^ However, the gold standard polysomnography is a complex and challenging exam,^7^ which increases the need for alternative markers to guide its indication. It is well known that sleep, which under normal physiological conditions must guarantee the body's regeneration through the parasympathetic system, is disturbed by frequent hypoxia, respiratory acidosis, and awakenings that activate the sympathetic system. In addition, OSAS is also reported as a cardiovascular risk factor, as there is a sympathetic tone increase due to repetitive apneas accompanied by hypoxia during sleep,^8^ which indicate, altogether, an association of sleep disorders and autonomic nervous system (ANS) alterations.

Although defective anionic transport through epithelial cells is accepted as a basic defect in CF, many characteristics observed in people with CF and affected organs are modulated by their nervous system.^9^ Several studies suggest that autonomic function is altered in individuals with CF, such as changes in heart rate variability (HRV), gastrointestinal motility, mucus secretion and bronchial smooth muscle contraction, controlled by the ANS, and that can favor the manifestations of the disease, leading to a worse prognosis.^10^ ^,^ ^11^ HRV is able to non-invasively assess changes in the function of the ANS, being an indicator of increased risk of cardiac mortality. Although HRV abnormalities identified in the CF population are still divergent in the literature,^12^ ^,^ ^13^ its use as an early marker of ANS alterations could be clinically useful to monitor several CF-related complications, including sleep disturbances, that are closely related to the autonomic tonus modulation.

Therefore, considering that HRV assessment is a simple and easy-to-perform method, the study of the relation between cardiovascular changes and the presence of sleep disorders is justified. Evidence obtained from clinical trials has shown increased activation of the sympathetic system, which also occurs in sleep apnea, when ANS dysregulation is observed.^14^ Thus, the present study aims to assess the association of sleep disorders with the findings of heart rate variability in children and adolescents with CF.

## METHOD

A cross-sectional study was carried out evaluating individuals diagnosed with CF confirmed by genetic testing, aged between six and 18 years. Patients in regular follow-up at the CF referral center (Hospital Infantil Nossa Senhora da Glória — HINSG) were invited to participate in the study. Those who met the selection criteria and whose parents or guardians authorized their participation by signing the informed consent form (ICF) were evaluated according to the experimental protocol, performed in two consecutive days. Patients with unstable clinical conditions or disease complications in the last 30 days and those who did not complete all tests were excluded from the sample. The study followed the criteria of the Ethics in Research with Human Beings, according to Resolution No. 466/12 of the Brazilian National Health Council, and was approved by the Research Ethics Committee of Pontifícia Universidade Católica do Rio Grande do Sul (PUCRS) under No. 2.459.354.

At first day, during consultation at the reference hospital, information was recorded to characterize the clinical and sociodemographic profile through interviews with those responsible and data collections in the medical records. After that, spirometry was performed to assess pulmonary function; bioimpedance was performed to assess the nutritional status. At the end of these procedures, patients were referred for evaluation of the autonomic nervous system (ANS) at rest. At the end of the day, participants were directed to perform a nocturnal polysomnography in a specialized clinic. The next day, in the morning, they returned to the clinic to finish and remove the equipment.

Regarding the study outcomes, the primary was the presence of sleep disorders and the secondary was the measurement of HRV. For sample calculation, the studies of Veronezi et al.^15^ and McNarry and Mackintosh^13^ were used, which evaluated, respectively, sleep-disordered breathing and HRV in patients with CF. Thus, using as reference variables the apnea and hypopnea index and the HRV RR intervals standard deviation (SDNN), as well as a power of 95%, significance level at 5%, and a correlation between variables of 0.5, a sample size of 30 individuals was estimated.

Sociodemographic data including age, sex, and city of origin were collected. Clinical data, such as type of mutation, presence of chronic airway colonization by *Pseudomonas Aeruginosa* ^16^ and Shwachman-Kulczycki (SK) score were collected from the medical records of the CF center. The classification of clinical manifestations was performed using the SK score, which generates a score based on aspects of nutrition, general activity, physical exams, and radiological findings. Each criterion varies from 5 to 25 points, in which the lower the score, the more severe the patient: excellent (86–100), good (71–85), medium (56–70), poor (41–55), and severe (≤40).^17^

Pulmonary function assessment was performed with a KoKo spirometer (nSpire Health, USA). The subjects were instructed to perform an exhalation, followed by a slow and deep inhalation, and then verbally encouraged to perform a maximum and forced exhalation. At least three maneuvers were conducted and accepted when the curves and their respective values were reproducible, with differences of less than 5% or 150mL between them, according to the criteria of the American Thoracic Society (ATS).^18^ The variables studied were forced vital capacity (FVC), FEV_1_, forced expiratory flow between 25 and 75% of forced vital capacity (FEF_25-75%_), and the FEV_1_/FVC ratio. In order to obtain the predicted values, the international equation of the 2012 Global Lung Function Initiative was used.^19^

For the assessment of nutritional status, bioimpedance was performed with the Inbody 720 equipment (InBody Co., Los Angeles, USA). Weight and body mass index (BMI) data were obtained, and the Z score was calculated according to the WHO Anthroplus software, version 1.0.4.^20^

Autonomic evaluation was performed with the RS800CX equipment (Polar Electro Oy Inc., Finland). This heart rate monitor provides valid measurements when compared to the echocardiogram^21^ and is reliable for the evaluation of HRV in children and adolescents.^22^ Data collection was performed in a room of controlled temperature; individuals remained in the supine position and were instructed to remain at rest, avoiding talking during the collection. The evaluation lasted 25 minutes, with the first five being eliminated to stabilize parameters. For analysis, data were analyzed using the Kubios HRV Standard Software, version 3.1.0 (HRV analysis, University of Eastern Finland).^22^ Only series with more than 95.0% of sinus rhythm were included in the study. The time and frequency domains were evaluated. In the time domain of variability we used: SDNN — standard deviation of all RR (inter-beat) intervals, expressed in ms; rMSSD — square root of the mean of squares of the successive differences between RR intervals greater than 50ms, expressed in ms; pNN50 — percentage of successive cycles that show differences in duration above 50ms, expressed as a percentage and geometric indices SD1 — instantaneous record index of beat-to-beat variability, representing the parasympathetic activity, whereas SD2 index reflects global variability. For the frequency domain, the following indices were evaluated: LF — low frequency; HF — high frequency; and LF/HF — low frequency/high frequency ratio. The SDNN index was normalized according to the reference by Gassior et al.^23^, which established normative values in schoolchildren, enabling normal and abnormal classification.

Baseline nocturnal polysomnography is a gold standard test that evaluates sleep stages. All participants underwent complete nocturnal baseline polysomnography, type II, performed at home, and equipment used was the Alice PDx by Philips. The assembly and preparation of the exam was performed in a specialized sleep laboratory, according to the recommendations of the American Academy of Sleep Medicine (AASM).^4^ Sleep stages were analyzed every 30 seconds or one series using the standard criterion, and the analysis of respiratory parameters was performed in periods of 120 seconds due to the slower occurrence of respiratory events. In each period, the following were analyzed: sleep phases, number of apneas and hypopneas, awakenings by electroencephalogram (EEG), and oxyhemoglobin desaturation. Obstructive apneas were defined when there was a decrease of more than 90% in the flow through the oronasal thermal flow sensor or alternative apnea flow sensor for at least two respiratory cycles and associated with respiratory effort during that period. Hypopneas were defined when there was a drop of more than 30% in the flow sensor of the nasal pressure transducer or alternative hypopnea alternative flow sensor, which lasted more than two respiratory cycles, and associated with a drop of more than 3% in SpO_2_ and/or awakening, being considered obstructive when associated with at least one of the following attributes: snoring, paradoxical thoracoabdominal movement, or flattening of the nasal transducer flow curve.^24^ Nocturnal hypoxemia was defined as 5% of sleep time with SpO_2_ below 90%. The OSAS was defined as an obstructive apnea and hypopnea index higher than two per hour.

Kolmogorov-Smirnov test was used to assess the normality of data. The results with symmetrical distribution were presented as mean and standard deviation, whereas asymmetric data were presented as median and interquartile range. Qualitative variables were presented in absolute and relative frequency. Fisher's exact test was used to evaluate associations. In order to correlate the HRV variables with sleep disturbance variables, Spearman's correlation test was used. The significance level adopted was p≤0.05 and the statistical program used was Stata, version 12.0.

## RESULTS

A total of 57 patients were invited to participate in the study. Of these, 21 refused, four patients presented with exacerbation, one was on oxygen therapy, and one patient did not complete the polysomnography, thus being excluded from the sample. The final sample consisted of 30 individuals, with a mean age of 11.2 years, most of whom were female (66.6%). SK score presented a mean of 86.2±13.4, characterizing most samples as excellent. As for pulmonary function, assessed with spirometry, the mean percentage of FEV_1_ and FVC was 62.8 and 78.6%, respectively (Table 1).

**Table 1:** Characterization of the study sample.

		n=30
Demographics		
	Age (years old)	11.2±3.7
	Female, n (%)	20 (66.6)
Anthropometric		
	Height (cm)	139.2±16.9
	Height for age (Z score)	-0.5 (-1.3–0.2)
	BMI (kg/m^2^)	16.6±2.6
	BMI (Z score)	-0.3 (-1.4–0.1)
	SMM (kg)	13.4±5.7
	FM (kg)	7.0±4.8
	Body fat (%)	19.6±7.6
Genotyping		
	F508del heterozygote (%)	43.3
Chronic airway colonization		
	*Pseudomonas aeruginosa*, n (%)	10 (33.3)
Pancreatic insufficiency		
	Yes, n (%)	27 (90.0)
	Shwachman-Kulczycki score	86.2±13.4
Lung function		
	FEV1 (L)	1.5±0.7
	FEV1 (%)	62.8±27.6
	FVC (L)	1.9±0.9
	FVC (%)	78.6±21.3
	FEF_25-75%_ (L/min)	1.4±0.8
	FEF_25-75%_ (%)	55.0±29.0
	FEV1/FVC	0.8±0.1

BMI: body mass index; SMM: skeletal muscle mass; FM: fat mass; FEV_1_: forced expiratory volume in the first second; L: liters; FVC: forced vital capacity; FEF_25-75_: forced expiratory flow between 25 and 75% of FVC. Data presented as mean and standard deviation or n (%), as indicated.

In the sleep assessment, reduced sleep efficiency was identified, with a mean of 66.5%, an awakening index of 8.7 (6.8–11.2), and a respiratory disturbance index with a median of 2.6 (1.9–3.9). The presence of OSAS was also identified in 30% of the sample. The median SpO_2_ was 95% (94–97), and the median minimum SpO_2_ was 89% (83–91), in addition to 26.7% of the patients with nocturnal hypoxemia. In the HRV analysis (time-domain) an average SDNN of 60.8±45.9 was found, whereas in the frequency-domain, a mean of 60.4±17.2 for the normalized LF and of 38.9±17.2 for the normalized HF were found, as described in Table 2.

**Table 2:** Main variables of nocturnal polysomnography and heart rate variability.

		n=30
Polysomnography		
	TTS (min)	451.2±71.2
	Sleep efficiency (%)	66.5±12.0
	Stage I (%)*	4.2 (2.8–5.9)
	Stage II (%)	45.0±10.5
	Stage III (%)	27.9±8.0
	REM sleep (%)	21.2±6.1
	Awake time (min)	130.0±68.6
	Awakenings (n˚)	73.7±38.4
	Awakening index (n˚/h)*	8.7 (6.8–11.2)
	Respiratory events (n)*	18.5 (14.0–30.8)
	Central apnea (n˚)	6.3±12.1
	Obstructive hypopnea (n˚)*	9.0 (6.0–15.5)
	RDI (n˚/h)*	2.5 (1.9–3.9)
	AHI (n˚/h)*	2.1 (1.3–3.9)
	OAHI (n˚/h)*	1.3 (1.0–2.2)
	Presence of OSAS (%)	30.0
	Mean SpO_2_ (%)*	95.0 (94–97)
	Minimum SpO_2_ (%)*	89.0 (83–91)
	% SpO_2_ time <90%*	0.3 (0.1–5.6)
Heart rate variability		
	SDNN	60.8±45.9
	rMSSD	59.9±61.3
	pNN50	22.4±20.6
	LF	2971.7±7962.6
	LF (nu)	60.4±17.2
	HF	2242.5±4602.5
	HF (nu)	38.9±17.2
	VLF	502.8±1463.5
	LF/HF	2.0±1.9
	SD1	43.8±42.7
	SD2	66.9±53.3

Data expressed as mean and standard deviation or

* median and interquartile range. TTS: total sleep time; REM: rapid eye movement; min: minute; RDI: respiratory disturbance index; AHI: apnea and hypopnea index; OAHI: obstructive apnea and hypopnea index; OSAS: obstructive sleep apnea syndrome; SpO_2_: peripheral oxyhemoglobin saturation; SDNN: standard deviation of all RR intervals; rMSSD: square root of the mean of the sum of squares of the differences between adjacent RR intervals; pNN50: number of pairs of adjacent RR intervals that differ by more than 50ms during recording; LF: low frequency; HF: high frequency; nu: normalized units; SD1: standard deviation of the instant beat-to-beat variability; SD2: long-term standard deviation of continuous RR intervals.

When correlating the variables of sleep and HRV, only a significant but weak correlation (r=0.35; p=0.052) between LF/HF and the minimum SpO_2_ during sleep (Table 3) could be found. However, when separating the sample in relation to lung function, individuals with FEV_1_<60% showed a significant strong correlation between the LF/HF index and the minimum SpO_2_ during sleep (r=0.71; p=0.020) (Table 4).

**Table 3:** Correlation between sleep variables and heart rate variability.

(n=30)	Sleep efficiency (%)		AHI (n˚/h)		OHAI (n˚/h)		Mean SpO_2_ (%)		Minimum SpO_2_ (%)		% TST SpO_2_<90%	
	rho	p-value*	rho	p-value*	rho	p-value*	rho	p-value*	rho	p-value*	rho	p-value*
SDNN	0.26	0.151	0.11	0.536	0.03	0.857	0.03	0.844	0.00	0.981	0.29	0.114
pNN50	0.17	0.341	0.06	0.721	-0.04	0.819	-0.14	0.451	-0.03	0.843	0.20	0.262
LF	0.16	0.386	-0.02	0.908	-0.10	0.582	-0.02	0.889	0.08	0.686	0.10	0.580
HF	0.20	0.284	0.05	0.774	-0.07	0.703	-0.04	0.790	-0.09	0.631	0.23	0.212
LF/HF	-0.19	0.302	-0.17	0.348	-0.00	0.970	0.01	0.948	0.35	0.052	-0.21	0.246

* Spearman's correlation test; TTS: total sleep time; HAI: hypopnea apnea index; OHAI: obstructive hypopnea apnea index; SpO_2_: peripheral oxyhemoglobin saturation; SDNN: standard deviation of all RR intervals; pNN50: number of pairs of adjacent RR intervals that differ by more than 50ms during recording; LF: low frequency; HF: high frequency; rho: coefficient of correlation.

**Table 4:** Correlations between sleep variables and heart rate variability in individuals with FEV_1_<60%.

(n=10)	Sleep variables									
	Mean SpO_2_ (%)		Minimum SpO_2_ (%)		%TST with SpO_2_<90%		HAI (n˚/h)		OHAI (n˚/h)	
	rho	p-value*	rho	p-value*	rho	p-value*	rho	p-value*	rho	p-value*
SDNN	-0.26	0.458	-0.42	0.218	0.27	0.433	-0.06	0.854	-0.05	0.879
pNN50	-0.16	0.645	-0.36	0.298	0.13	0.712	-0.04	0.907	-0.17	0.622
LF	-0.25	0.480	-0.32	0.362	0.14	0.687	0.01	0.960	-0.01	0.959
HF	-0.43	0.205	-0.58	0.075	0.48	0.160	0.13	0.700	0.14	0.697
LF/HF	0.43	0.212	0.71	0.020	-0.59	0.069	-0.11	0.751	-0.15	0.672

* Spearman's correlation test; SpO_2_: peripheral oxyhemoglobin saturation; TST: total sleep time; HAI: hypopnea apnea index; OHAI: obstructive hypopnea apnea index; SDNN: standard deviation of all RR intervals; pNN50: number of pairs of adjacent RR intervals that differ by more than 50ms during recording; LF: low frequency; HF: high frequency; rho: coefficient of correlation.

Table 5 shows the comparison of SDNN (normal or altered), nocturnal hypoxemia and the presence of OSAS between patients with FEV_1_ below or above 60%. The results indicate that patients with FEV_1_<60% had a higher prevalence of altered SDNN (p=0.032) and nocturnal hypoxemia (p=0.032). No differences were found for OSAS (p=0.082).

**Table 5:** Prevalence of altered heart rate variability (standard deviation of all) and sleep disturbances (nocturnal hypoxemia and obstructive sleep apnea syndrome), according to lung function.

		FEV_1_≥60%	FEV_1_<60%	p-value
		n (%)		
SDNN				
	Normal	18 (85.7)	4 (44.4)	0.032*
	Altered	3 (14.3)	5 (55.6)	
Nocturnal hypoxemia				
	No	18 (85.7)	4 (44.4)	0.032*
	Yes	3 (14.3)	5 (55.6)	
OSAS				
	No	17 (81.0)	4 (44.4)	0.082
	Yes	4 (19.0)	5 (55.6)	

* Fisher's exact test. FEV_1_: forced expiratory volume in the first second; SDNN: standard deviation of all RR intervals; OSAS: obstructive sleep apnea syndrome.

## DISCUSSION

The results of the present study suggest that there is a significant correlation between sleep disorder and HRV only in patients with more severe disease (pulmonary function of less than 60%), since no significant correlations were found in the total sample. Previous studies have shown that sleep disorders were associated with an increase in the sympathetic modulation, especially respiratory disorders.^8^ However, this appears to be the first study that evaluated the association of resting HRV with sleep disorders in children and adolescents with CF.

In the sleep assessment, reduced sleep efficiency (66.5%) and a wake-up rate of 9.8 was identified. These findings are in accordance with data in the literature that demonstrate reduced sleep efficiency and a positive correlation with disease severity.^2^ Nocturnal hypoxemia was present in 26.7% of individuals; evidence suggests that frequent episodes can lead to inflammation of the pulmonary parenchyma, as well as the development of pulmonary hypertension and failure of the right ventricle, leading to associated changes in HRV, with increased sympathetic predominance.^24^

Regarding the HRV data, the SDNN and RMSSD indices of children and adolescents with CF during rest are also equivalent to those of obese children, based on the study by Plaza-Florido et al.,^25^ representing a low vagal predominance. The SDNN index, of vagal modulation, in children and adolescents with CF is like that of heart transplanted children, as shown in the study by Williams et al.^26^ These data are in agreement with those found in the present sample, indicating low parasympathetic modulation.

No significant correlation between sleep variables and HRV in the total sample was found, only in individuals with lower pulmonary function (FEV_1_<60%), who showed a significant correlation between the LF/HF index and the minimum SpO_2_ during sleep (r=0.71). The LF/HF ratio reflects the balance between sympathetic and parasympathetic activity, which may indicate changes in HRV,^14^ since individuals with better pulmonary function had a higher mean in this index. Another study demonstrated the importance of the LF/HF ratio by showing that it correlates with the presence of OSAS.^8^ The increase in this index in individuals with worse pulmonary function indicates a tendency of alterations in the ANS.^27^ However, there are no studies available in CF patients that have assessed this association, making it difficult to discuss the data. On the other hand, this relation has been shown to be increased in situations in which the body needs to compensate for stress events, seeking to maintain homeostasis, as in chronic diseases, in which this compensation occurs for a prolonged period, as well as the effects on the ANS.^28^ An increase was identified in patients with chronic obstructive pulmonary disease (COPD), indicating a worse prognosis, as well as in diabetic patients, related to a higher risk of cerebral and cardiovascular events.^29^

Previous studies have shown that sleep-disordered breathing was associated with an increase in sympathetic modulation.^6^ In the present study, the SRD index presented a median of 2.6 (1.9–3.9). When the SDNN was normalized and classified as normal and altered, according to Gasior et al.^23^ patients who presented FEV_1_<60% had a higher prevalence of altered SDNN and nocturnal hypoxemia. Recurrent episodes of hypoxia and awakenings during sleep lead to ANS dysregulation, whereas the domain of the sympathetic nervous system in sleep apnea leads to the development of cardiovascular diseases. This corroborates with another study in children aged between three and 12, which concluded that sleep-disordered breathing with frequent hypoxia disrupt the maturation of the ANS.^30^ On the other hand, Urbanik et al.^14^ demonstrated that patients with OSAS had reduced HRV in the time-domain and spectral parameters, and that these changes occur not only during sleep, but also during daily activity. Data herein demonstrated the presence of OSAS in 30% of the sample.

The present study has some limitations, including its small sample size for analysis of subgroups and its cross-sectional design, which does not allow causal inference with the data obtained. However, the results presented can contribute to the characterization of sleep disorders and changes in HRV in children and adolescents with CF, allowing a preliminary analysis of their association. Selection of the sample by convenience, age range between six and 18 years old, and OSAS criteria may also be considered as limitations.

In conclusion, the results of the present study indicate a weak correlation of sleep disorders (minimum SpO_2_) with HRV parameters (LH/HF) in children and adolescents with CF. When pulmonary function was reduced, a stronger correlation was found, highlighting the influence of disease severity. In addition, these patients have a higher prevalence of ANS disorders, nocturnal hypoxemia, and presence of OSAS. Considering that the present study presents preliminary descriptive data, the possible use of HRV measures as markers of sleep disorders still needs to be better understood in future studies.
